# TRPV1 mediates astrocyte activation and interleukin-1β release induced by hypoxic ischemia (HI)

**DOI:** 10.1186/s12974-019-1487-3

**Published:** 2019-05-29

**Authors:** Xing-Liang Yang, Xin Wang, Lin Shao, Guang-Tong Jiang, Jia-Wei Min, Xi-Yu Mei, Xiao-Hua He, Wan-Hong Liu, Wen-Xian Huang, Bi-Wen Peng

**Affiliations:** 1 Department of Physiology, Hubei Provincial Key Laboratory of Developmentally Originated Disorder, School of Basic Medical Sciences, Wuhan University, Donghu Rd185#, Wuhan, 430071 Hubei China; 2 No.1 Middle School affiliated to Central China Normal University, Wuhan, China; 3 Department of Pathophysiology, School of Basic Medical Sciences, Wuhan University, Wuhan, China; 4 Department of Immunology, School of Basic Medical Sciences, Wuhan University, Wuhan, China; 5 Department of Pathology, Renmin Hospital of Wuhan University, Jiefang Rd238#, Wuhan, 430071 Hubei China

**Keywords:** HI, Astrocyte, TRPV1, IL-1β

## Abstract

**Background:**

Hypoxic-ischemic encephalopathy (HIE) is a serious birth complication with high incidence in both advanced and developing countries. Children surviving from HIE often have severe long-term sequela including cerebral palsy, epilepsy, and cognitive disabilities. The severity of HIE in infants is tightly associated with increased IL-1β expression and astrocyte activation which was regulated by transient receptor potential vanilloid 1 (TRPV1), a non-selective cation channel in the TRP family.

**Methods:**

Neonatal hypoxic ischemia (HI) and oxygen-glucose deprivation (OGD) were used to simulate HIE in vivo and in vitro. Primarily cultured astrocytes were used for investigating the expression of glial fibrillary acidic protein (GFAP), IL-1β, Janus kinase 2 (JAK2), signal transducer and activator of transcription 3 (STAT3), and activation of the nucleotide-binding, oligomerization domain (NOD)-like receptor pyrin domain-containing protein 3 (NLRP3) inflammasome by using Western blot, q-PCR, and immunofluorescence. Brain atrophy, infarct size, and neurobehavioral disorders were evaluated by Nissl staining, 2,3,5-triphenyltetrazolium chloride monohydrate (TTC) staining and neurobehavioral tests (geotaxis reflex, cliff aversion reaction, and grip test) individually.

**Results:**

Astrocytes were overactivated after neonatal HI and OGD challenge. The number of activated astrocytes, the expression level of IL-1β, brain atrophy, and shrinking infarct size were all downregulated in TRPV1 KO mice. TRPV1 deficiency in astrocytes attenuated the expression of GFAP and IL-1β by reducing phosphorylation of JAK2 and STAT3. Meanwhile, IL-1β release was significantly reduced in TRPV1 deficiency astrocytes by inhibiting activation of NLRP3 inflammasome. Additionally, neonatal HI-induced neurobehavioral disorders were significantly improved in the TRPV1 KO mice.

**Conclusions:**

TRPV1 promotes activation of astrocytes and release of astrocyte-derived IL-1β mainly via JAK2-STAT3 signaling and activation of the NLRP3 inflammasome. Our findings provide mechanistic insights into TRPV1-mediated brain damage and neurobehavioral disorders caused by neonatal HI and potentially identify astrocytic TRPV1 as a novel therapeutic target for treating HIE in the subacute stages (24 h).

**Electronic supplementary material:**

The online version of this article (10.1186/s12974-019-1487-3) contains supplementary material, which is available to authorized users.

## Introduction

HIE resulting from oxygen deprivation and blood flow reduction to the brain is a common cause of brain damage [1]. The mechanisms involved in the brain damage include excitotoxicity, apoptosis, and glial overactivation [2]. Although the importance of microglial activation in hypoxia-induced neuroinflammation is well explored [3], the role of astrocyte activation in this process has attracted more attention. It has been explored that activation of astrocytes under pathological conditions [4], such as ischemic damage [5] and neuroinflammation [6–8], are closely related to the pathogenesis of brain injury [9]. Activation of astrocytes and subsequent increase of IL-1β release are shown to be a key determinant of the outcome and prognosis of HIE [10–12]. Astrocytes are the predominant source of IL-1β in post-traumatic stress disorder [13] and prion disease [14]. Active NLRP3 inflammasome, which cleaves the pro-inflammatory cytokines IL-1β to its active form [15], has been detected in astrocytes in several animal models of intracerebral hemorrhage, such as ischemia/reperfusion injury and hemorrhage transgenic amyotrophic lateral sclerosis [16–19]. Moreover, inhibiting the activation of NLRP3 inflammasome attenuates neonatal HI-induced brain injury in rats [20, 21], and systemic neutralizing IL-1β by infusions of anti-interleukin-1β antibodies reduces short-term brain injury after cerebral ischemia in the ovine fetus [22].

TRPV1 channel is the prototypic member of the TRP family and displays dynamic ion selectivity for ions including H^+^, Na^+^, Ca^2+^, and Mg^2+^ [23]. In the CNS, TRPV1 participates in synaptic transmission, neurogenesis, and neuroinflammation [24, 25]. Recent studies suggest that TRPV1 is functionally expressed in CNS glial cells, especially microglia and astrocytes [26, 27]. In astrocytes, TRPV1 activation mediates its migration, chemotaxis, and the expression of GFAP, during stress and injury [28]. TRPV1 activation stimulates a JAK2-STAT3 pathway to regulate astrocyte activation, astrocyte hypertrophy, and the expression of cytokines such as IL-1β and IL-6 in vitro and in vivo [29–32]. TRPV1 activation participated in neonatal brain injuries [33, 34], and overactivated astrocytes exacerbate neurological deficits [35]. These findings strongly suggest that TRPV1 may mediate astrocytic activation and IL-1β release in HIE.

We have previously reported that TRPV1 promoted the activation of microglia and upregulated the expression of TNF, IL-1β, IL-6, and high mobility group box 1 (HMGB1) in repetitive febrile seizure (FS) mice [36]. The main purpose of the present study was to determine whether TRPV1 aggravated brain damages, neurological deficits by promoting astrocytes activation, and neuroinflammation in a mouse model of neonatal HI. We found that in the subacute stage (24 h), TRPV1 activation increases the phosphorylation of JAK2 and STAT3, which results in an increased expression of GFAP and IL-1β. TRPV1 activation promotes the activation of the NLRP3 inflammasome, which drives the release of IL-1β. Furthermore, knocking out TRPV1 significantly improves neonatal HI-induced neurobehavioral disorders.

## Materials and methods

### Animal models and treatments

B6.129X1-TRPV1 KO mice used in experiments were from the Jackson Laboratory, and wild-type (WT) mice were provided by the Hubei Province Center for Animal Experiments. Animal experiments were approved by the Care and Use Committee of Wuhan University Medical School. B6.129X1-TRPV1 KO mice and wild-type mice were both on a C57BL/6J background. Mice were grouped randomly and kept on a 12-h light–dark cycle, in a room maintained at 25 ± 2 °C and relative humidity of 60~80%. All mice were housed at the animal facility of the Animal Biosafety Level III Laboratory (ABSL-III) of the Wuhan University. The day of the birth was defined as postnatal day zero (P0). Mice pups (*n* = 160) of P9 were used in this study and randomly divided into the five groups (*n* = 32 each group): WT-Sham, WT-HI, WT-Capsaicin-HI (intraperitoneal injection of 3 mg/kg capsaicin 0.5 h before neonatal HI), KO-Sham, and KO-HI.

### Neonatal hypoxia-ischemia model

A well-characterized model of neonatal HI was followed as a previously described [2, 37]. P9 C57BL/6 WT and TRPV1 KO mice of both genders (5 ± 1 g of body weight, equal males, and females were chosen for each group) were anesthetized by inhalation of isoflurane. The sterilized skin was incised with an ophthalmology scissor. And the right pulsating carotid artery was carefully separated. The upper and lower ends of the right carotid artery were tied using 4-0 surgical suture before cutting the artery in the middle. The skin incision was sutured with the same surgical suture. All the above experimental surgical instruments were sterilized. After 2 h of recovery, the pups were placed in an airtight transparent chamber, and the chamber was placed into a 37 °C incubator to maintain a constant thermal environment. The pups were maintained in 8% O_2_ in N_2_ for 45 min. After the hypoxic process, the mouse pups were put back in the cages. Successful HI model showed significant edema in the ipsilateral hemisphere, while the sham group (underwent anesthesia, neck incision, and suture) did not. The mortality of this model was about 10%.

### Nissl staining

We followed detailed protocols that have been previously reported [38]. Briefly, at 24 h after neonatal HI, mice pups were perfused with phosphate-buffered saline (PBS) followed by 4% paraformaldehyde (PFA). The brains were then obtained for Nissl staining following the standard protocol.

### Infarct volume measurement

The procedure has been previously described [39]. At 24 h post-HI, animals were transcardially perfused with PBS under deep anesthesia. The brains were obtained and sectioned into 2 mm slices and then immersed in 2% 2, 3, 5-triphenyltetrazolium chloride monohydrate (TTC) solution at 37 °C for 10 min, followed by 4% PFA to completely fix the tissue. The infarct volume was traced and analyzed by ImageJ software (version 1.41).

### Primary mouse cortical astrocytes cultures

Astrocyte culture was prepared as previously described [40]. P0 mice were euthanized after being disinfected with 75% ethanol. The brain tissue was isolated and then put into the pre-cooled PBS. In order to obtain dissociated cells, meninges were removed and the clean cerebral cortex was digested in Hank’s balanced salt solution (HBSS) containing 0.125% trypsin at 37 °C for 10 min. Complete growth media (1× Dulbecco’s modified Eagle’s medium (DMEM)/F12, 10% heat-inactivated fetal bovine serum, 1% l-glutamine, and 1% penicillin/streptomycin) were added to terminate the digestion. Gently blow digested tissues with pap dropper and then centrifuge at 1000 rpm for 5 min. Dissociated cells were planted in T75 flasks in glial medium and then maintain at conventional cell culture incubator (37 °C, 5% CO_2_) for 10–12 days. To purify astrocytes from microglia and oligodendrocytes, the cells in the T75 were subjected to continuous shaking at 37 °C (200 rpm, 10 h). The remaining cells were identified by immunofluorescence staining with anti-GFAP antibody, and approximately 95% of the remaining cells were GFAP-positive (data not shown).

### OGD progression

Oxygen-glucose deprivation (OGD) was conducted as described previously [41]. Briefly, astrocytes were grown in complete growth media for 9 days as monolayers in cell culture incubator (95% O_2_ and 5% CO_2_ at 37 °C). And all groups of cell medium were changed to serum-free growth medium 24 h before OGD. To initiate OGD in vitro, the plates were washed with PBS three times, added OGD medium (serum- and glucose-free DMEM), and placed in a hypoxic/anoxic chamber (1% O_2_, 5% CO_2_, and 94% N2 at 37 °C); 3.5 h later, plates were removed from the anaerobic chamber and OGD medium was changed to complete growth medium. At the same time, control glucose-containing cultures remained in a regular incubator (5% CO_2_ and 95% O_2_) and the medium was changed to fresh complete growth medium with OGD group. The supernatants and cell extracts were collected after OGD for the following experiments.

### Real-time PCR

Total RNA was extracted from the cortical-derived astrocytes of WT and TRPV1 KO mice from different groups using TRIzol reagent (Invitrogen Life Technologies Corporation, USA) to detect the mRNA level of IL-1β, IL-6, TNF, IL-10, NLRP3, an apoptosis-associated speck-like protein containing carboxyl-terminal CARD (ASC), and caspase-1.

The quantity of total RNA was measured by a UV spectrophotometer (Biochrom Ltd., UK). Next, reverse transcription was performed using a cDNA synthesis kit (TaKaRa Biotechnology). QPCR was performed on a SYBR-Green premix (TransGen Biotech) according to the manufacturer’s specification with the primers listed in Table 1 (Sangon Biotech, Shanghai, Co., Ltd.). The cycling parameters for the CFX96 sequence detection system were 95 °C for 5 min; 40 cycles of 95 °C for 15 s, 58 °C for 30 s, and 72 °C for 20 s. The expression of target genes was normalized to the mRNA level of β-actin as an internal control. The ΔΔCt values of each group were analyzed, and the mRNA expression of different groups was normalized to 2^−ΔΔCt^.

**Table 1:** q-PCR primer sequences applied

Primer	Forward primer 5′-3′	Reverse primer 5′-3
NLRP3	ACGAGTCCTGGTGACTTTGTAT	TAGGTCCACACAGAAAGTTCTCTTA
Caspase1	ATGCCGTGGAGAGAAACAAGG	CCCCTGACAGGATGTCTCCA
ASC	ACGAGTCCTGGTGACTTTGTAT	TAGGTCCACACAGAAAGTTCTCTTA
IL-1β	TAAAGACCTCTATGCCAACACAGT	CTGACTTGGCAGAGGACAAAG
IL-6	TAGTCCTTCCTACCCCAATTTCC	TTGGTCCTTAGCCACTACTTC
TNF	CCAACAAGGAGGAGAAGTTCC	CTCTGCTTGGTGGTTTGCTAC
IL-10	TACTGCTAACCGACTCCTTAATGC	AGAAATCGATGACAGCGCCTC
β-actin	CACGATGGAGGGGCCGGACTCATC	TAAAGACCTCTATGCCAACACAGT

### Western blot analysis

Western blotting was performed according to the manufacturer’s specification. The brain tissue samples and astrocytes were collected after neonatal HI and OGD. Proteins were extracted by homogenizing in RIPA buffer (Santa Cruz Biotechnology, Santa Cruz, CA, USA) with phenylmethanesulfonyl fluoride (PMSF) and further centrifuged at 12,000 rpm at 4 °C for 10 min. The protein concentrations were measured using a detergent-compatible assay (Bio-Rad, Dc protein assay). Equal amounts of protein were loaded on an SDS-PAGE gel. After electrophoresis and transfer to a polyvinylidene fluoride (PVDF) membrane, the membranes were blocked by 5% skimmed milk for 3 h. The membranes were incubated with the GFAP, JAK2, and STAT3 antibody overnight at 4 °C. Details for the primary and secondary antibodies are listed in Table 2. After incubation, the membranes were washed at least three times with TBST (TBS containing 0.2% Tween-20) and were then incubated for 1.5 h with secondary antibodies at room temperature. The membranes were washed again with TBST three times. Finally, the reaction was developed using a chemiluminescent reagent (ECL; Ecl Advantage Inc., Menlo Park, California, USA) and the bands of different proteins were detected using an imaging densitometer (Bio-Rad, Foster City, CA, USA). The data were analyzed using ImageJ software (version 1.41).

**Table 2:** Antibodies for Western blot

Antibody	Host	Company	Cat. no.	Dilution	Duration
GFAP	Mouse	Cell signaling	#3670	1:1000	Overnight 4 °C
JAK2	Rabbit	Cell signaling	#3230	1:1000	Overnight 4 °C
p-JAK2	Rabbit	Abcam	ab32101	1:2000	Overnight 4 °C
STAT3	Rabbit	Hangzhou HuaAn Biotech	ET1607-38	1:1000	Overnight 4 °C
p-STAT3	Rabbit	Hangzhou HuaAn Biotech	ET1603-40	1:1000	Overnight 4 °C
β-actin	Mouse	Protein tech	HRP-60008	1:10000	Overnight 4 °C
Anti-mouse IgG-HRP	Goat	PMK Biotech	PMK-014-091	1:100000	2 h RT
Anti-rabbit IgG-HRP	Goat	PMK Biotech	PMK-014-090	1:100000	2 h RT

### Measurement of cytokine by enzyme-linked immunosorbent assay (ELISA)

The amounts of IL-1β in the culture supernatants in each group of astrocytes were measured by ELISA kit (purchased from 4A Biotech Co., Ltd) according to the manufacturer’s instructions. The serum samples of animals or cells from different groups were added into a 96-well plate. Then, it was incubated with the corresponding primary antibody followed for 90 min at 37 °C. After washing three times with wash buffer, the secondary antibody was added into the plate and incubated for 60 min at 37 °C. And then the absorbance at 450 nm (Thermo, USA) was recorded. The concentration of the target protein was calculated according to the standard curve and normalized to the total protein of the samples. Assays were performed independently three times using triplicate wells.

### Cell viability assay

Cell counting kit-8 (CCK-8) assay was performed as previously described [42]. Astrocytes were seeded at a density of 3 × 10^4^ mL^−1^ in 96-well plates. Cell viability was subsequently assessed using the CCK-8 (Dojindo, Tokyo, Japan) according to the manufacturer’s instructions. The optical absorbance at 450 nm was detected using a plate reader (Thermo Fisher Scientific).

### Immunofluorescence

Immunofluorescence staining was used to detect GFAP and IL-1β expression in peri-ischemic brain tissue, assembly of ASC speck and NLRP3 and caspase-1 expression in astrocytes.

Mice in each group were deeply anesthetized with 10% chloral hydrate and transcardially perfused first with PBS and then fixed in 4% paraformaldehyde solution at room temperature, dehydrated 24 h after HI. Serial coronal sections (5-μm thick with injury epicenter located centrally) prepared with cryotome (Leica, Wetzlar, Germany) were used for immunofluorescence labeling. Sections were incubated with 5% BSA for 30 min at 37 °C. The tissue slices were then incubated overnight with the anti-GFAP and anti-IL-1β antibodies. For the assessment of assembly of ASC speck and NLRP3 and caspase-1 expression in astrocytes, cells were fixed with methanol, washed with PBS three times, and incubated at 4 °C with anti-ASC, anti-NLRP3, and anti-caspase-1 antibodies. On the following day, the sections and cells were washed and subsequently incubated with secondary antibodies Cy3-conjugated Goat Anti-Rabbit IgG (H + L) and Alexa Fluor® 488 Conjugates Goat Anti-Mouse for 1 h at 37 °C. Dilutions for antibodies were listed in Table 3. Images obtained using a confocal microscope (Leica-LCS-SP8-STED). And images area and intensity were measured in ImageJ.

**Table 3:** Antibodies for immunohistochemistry and fluorescence staining

Antibody	Host	Company	Cat. no.	Dilution	Applied	Duration
GFAP	Mouse	Cell signaling	#3670	1:300	ICC	Overnight 4 °C
Iba1	Goat	NOVUS	NB100-1028	1:200	ICC	Overnight 4 °C
NRLP3	Rabbit	NOVUS	NBP2-12446	1:50	ICC	Overnight 4 °C
ASC	Mouse	Santa Cruz Bio.	SC-271054	1:50	ICC	Overnight 4 °C
Caspase-1	Mouse	Santa Cruz Bio.	SC-398715	1:50	ICC	Overnight 4 °C
Il-1β	Rabbit	Bio-Swamp	PAB30679	1:50	ICC	Overnight 4 °C
Anti-rabbit IgG-Cy3	Goat	Protein tech	SA00009-2	1:50	ICC	1 h 37 °C
Anti-mouse IgG-488	Goat	EarthOx	E032210-01	1:200	ICC	1 h 37 °C
DAPI		Beyotime	C1002	1;2000	ICC,IHC	1 min RT

### Morphological assessment

The morphological assessment of astrocytes after OGD was performed according to M. Neal [43]. Briefly, 20 × images (Olympus U-HGLGPS) were imported into ImageJ for morphological analysis. The glial processes could be analyzed using an ImageJ plugin named Analyze Skeleton [44]. The image was converted to binary and then skeletonized in ImageJ software, and then, the skeletonized image was put into the Analyze Skeleton plugin to get detailed information about the branch length of each cell. Cell soma size was measured by freehand selection outlining each cell soma and then selecting the “area” analyzes to measure. At least three fields were used for each section, and then, three biological replicates were used for each experiment. At least 20 cells were measured for each of the morphology assessments.

### Neurobehavioral testing

Cliff aversion reaction, Geotaxis reflex and Grip test were respectively assessed at 1 day (P10), 3 days (P12), and 7 days (P16) after neonatal HI. All of the above experiments were observed by at least two uninformed laboratory workers, and all groups of animals maintained the same test method.

Cliff aversion reaction was used to assess the ability of rodents’ respond to adverse environments [45]. Put two planks together and the board was 30 cm above the ground. The head and front upper limbs of the mouse were on the first plank, the rest of the body was on the second plank, and all the mice were placed in this position. When the mouse relaxed, the first plank was quickly lowered so that the bilateral forelimbs of the mouse were suddenly suspended. After a short delay, the mouse can return to the second plank. The time required for the mouse to withdraw the forelimb was examined. Each mouse was tested three times. If the forelimbs were not withdrawn for more than 60 s, it would be recorded as 60 s. Grip test was used to assess the performance and tolerance of mice [46]. The bilateral forelimbs of the mice were grasped on a metal wire (1.5 mm in diameter), the metal rope was 50 cm from the ground, and the ground was covered with cork. Record the time from the mouse holding the metal rope to releasing the metal rope and test each mouse three times.

Geotaxis reflex was considered to diagnose the function of vestibular and proprioception of mice [47]. A wooden board was placed at an angle of 45°, and then, the mouse was placed at the middle of the wood heading toward the lower end of the wood. The time required for the mouse to turn 180° toward the upper end of the board was recorded. One mouse was tested three times. If the time was more than 60 s, it would be regarded as the maximum value of 60 s.

### Experimental design and statistical analysis

Prism 7 (RRID: SCR_002798) software was used to analyze data and form the graphs in this work (including which tests were performed, exact *P* values, and sample sizes). Simply, one-way ANOVA with a test for linear trend, Tukey test was used as appropriate to analyze parametric statistics. At least three independent experiments were applied to collect effective data. Bias was avoided by making sure that assessor was blinded to collecting and analyzing data. *P* < 0.05 was considered significant. Average values represent the mean ± SEM. In the quantitative analysis of Fig. 5e–g, the number of animals used in neurobehavioral testing was indicated in figure legends. ^*^P represents the significance compared to sham or control. ^#^*P* represents the significance compared to WT-HI or WT-OGD.

## Results

### TRPV1 deficiency prevents the upregulation of GFAP expression induced by neonatal HI

**Fig. 1:**
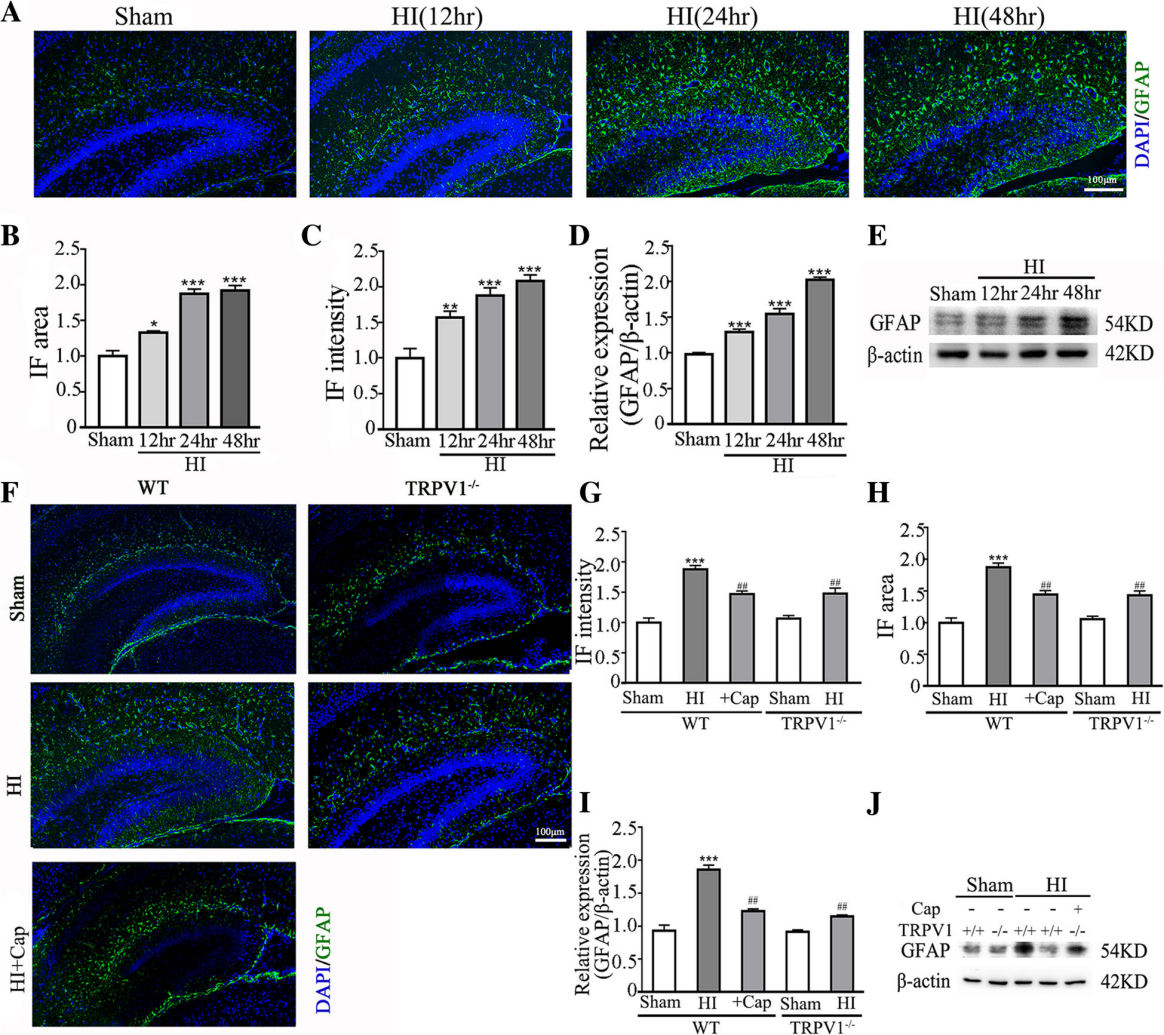
Knocking out TRPV1 inhibited GFAP expression in hypoxia-ischemia brain tissue. **a** After HI, GFAP expression was upregulated at 12 h, 24 h, and 48 h. GFAP expression levels by immunofluorescence were examined in the ipsilateral hemisphere sections from sham and HI groups. Scale bar = 100 μm. **b**, **c** Immunofluorescence intensity and cluster size of GFAP in the ipsilateral hemisphere hippocampus significantly increased in HI brain compared to sham brain. *n* = 6 for each group. **d**, **e** Western blot revealed upregulated expression of GFAP at 12 h, 24 h, and 48 h. GFAP expression levels were examined in ipsilateral hemisphere sections from Sham and HI. Quantification of independent blots. *n* = 5 for each group. **f** Knocking out TRPV1 or pretreating capsaicin reduced the upregulation of GFAP as shown by immunofluorescence at 24 h. Scale bar = 100 μm. **g**, **h** Knocking out TRPV1 or pretreating capsaicin reduced immunofluorescence intensity and cluster size of GFAP compared to HI group. *n* = 6 for each group. **i**, **j** Western blot revealed that knocking out TRPV1 or pretreating capsaicin reduced GFAP upregulation compared to HI group. Quantification of independent blots. *n* = 5 for each group. Cap, 3 mg/kg capsaicin. Average values represent the mean ± SEM. *P < 0.05, **P < 0.01, ***P < 0.001 versus sham (Tukey’s test after one-way ANOVA). ^##^*P* < 0.001 versus WT-HI (Tukey’s test after one-way ANOVA)

To determine whether TRPV1 mediates astrocyte activation following neonatal HI insult, the expression of GFAP were investigated after neonatal HI in both TRPV1 KO mice and congenic WT C57BL/6 mice. Immunofluorescence staining revealed significantly stronger labeling of GFAP in the ipsilateral hippocampus at 12 h, 24 h, and 48 h after neonatal HI compared to the sham group (Fig. 1a and Additional file 1: Figure S1A), and its expression saturated at 24 h (Fig. 1b, c). The labeling of Iba1 increased at 6 h, 12 h, and 24 h (Additional file 1: Figure S1A). Western blot results (Fig. 1d, e) showed that GFAP protein level was gradually upregulated at 12 h, 24 h, and 48 h after neonatal HI compared with the sham group, suggesting that HI insult significantly increases the number of activated astrocytes in the hippocampus.

On the other hand, immunofluorescence (Fig. 1f–h) and Western blot results (Fig. 1i, j) showed that the neonatal HI-induced increase of GFAP expression was significantly reduced at 24 h in the TRPV1 KO mice compared with WT mice. Consistent with the genetic ablation result, systemic desensitization of TRPV1 (intraperitoneal injection of 3 mg/kg capsaicin 0.5 h before neonatal HI) significantly reduced the increase of GFAP expression caused by neonatal HI in WT mice [48–50]. Knocking out TRPV1 mildly reduced Iba1^+^ cell, while intensely reduced GFAP^+^ cell in the ipsilateral hemisphere hippocampus (Additional file 1: Figure S1). Taken together, these results suggest that either genetic ablation of TRPV1 or systemic desensitization of TRPV1 effectively suppress the upregulation of GFAP expression induced by neonatal HI, highlighting the importance of TRPV1 in neonatal HI-induced astrocyte activation.

### Activation of astrocytes and increased expression of inflammatory cytokines induced by OGD are TRPV1-dependent

**Fig. 2:**
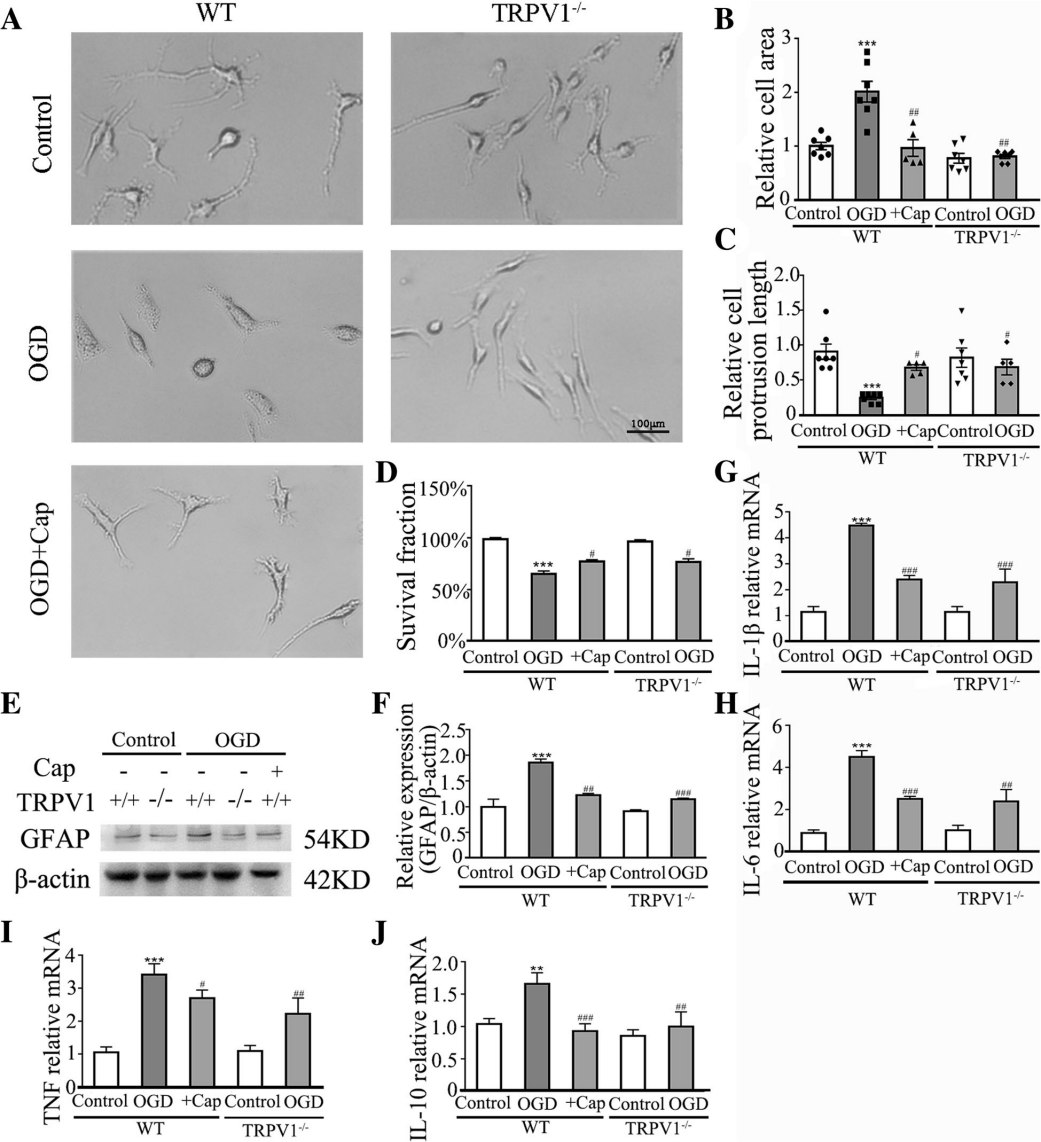
Knocking out TRPV1-protected astrocyte against ischemia-induced damage and inhibits its activation and expression of inflammatory cytokines. **a** Knocking out TRPV1 and pretreating with capsaicin (10 μM) 0.5 h before OGD prevented astrocytes from cell body edema, reduction of branch processes after OGD. **b**, **c** Cell area and protrusion length were normalized to control for other groups 24 h after OGD. *n* = 5–7 for each group. **d** Cell viability of astrocytes was promoted in the TRPV1^−/−^ and pre-treating with capsaicin group compared to OGD group. *n* = 6 for each group. **e** Western blot revealed that knocking out TRPV1 or pretreating capsaicin reduced the upregulation of GFAP compared to OGD group. **f** Quantification of independent blots. *n* = 5 for each group. **g**–**j** The graphical representation of the fold changes of the expression of IL-1β, IL-6, TNF, and IL-10. Knocking out TRPV1 or pre-treating with capsaicin inhibited the upregulation of IL-1β, IL-6, TNF, and IL-10 compared to OGD group. *n* = 5 for each group. Cap, 10 μM capsaicin. Average values represent the mean ± SEM. **P < 0.01, ***P < 0.001 versus control (Tukey’s test after two-way ANOVA). ^#^*P* < 0.05, ^##^*P* < 0.01, ^###^*P* < 0.001 versus WT-OGD (Tukey’s test after one-way ANOVA)

To explore the role of TRPV1 on HI-induced activation of astrocytes in vitro, hippocampal astrocytes were isolated and cultured to undergo OGD. Either TRPV1 deficiency or capsaicin-induced TRPV1 desensitization, pretreating with capsaicin (10 μM) 0.5 h before OGD, prevented astrocytes from cellular edema (Fig. 2a, b), reduction of branch processes (Fig. 2a, c), and cell death (Fig. 2d) at 24 h after OGD. In addition, the upregulation of GFAP reduced in TRPV1 deficiency astrocytes and WT capsaicin pretreatment astrocytes (Fig. 2e, f).

Activated astrocytes release several cytokines in neurological diseases [9]. OGD challenge significantly increased IL-1β (Fig. 2g), IL-6 (Fig. 2h), and TNF (Fig. 2i) mRNA expression level, which was inhibited by genetic ablation and capsaicin-induced desensitization of TRPV1 (Fig. 2g–i). Of note, although the expression level of the anti-inflammatory cytokine (IL-10) increased after OGD, genetic ablation and capsaicin-induced desensitization of TRPV1 only slightly inhibited the upregulation (Fig. 2j). These results suggested that TRPV1 is required for astrocyte activation, morphological change, and the expression of pro-inflammatory cytokines, especially IL-1β and IL-6 in vitro.

### Activation of JAK2-STAT3 signaling in astrocytes is required for OGD-induced TRPV1-dependent expression of IL-1β

**Fig. 3:**
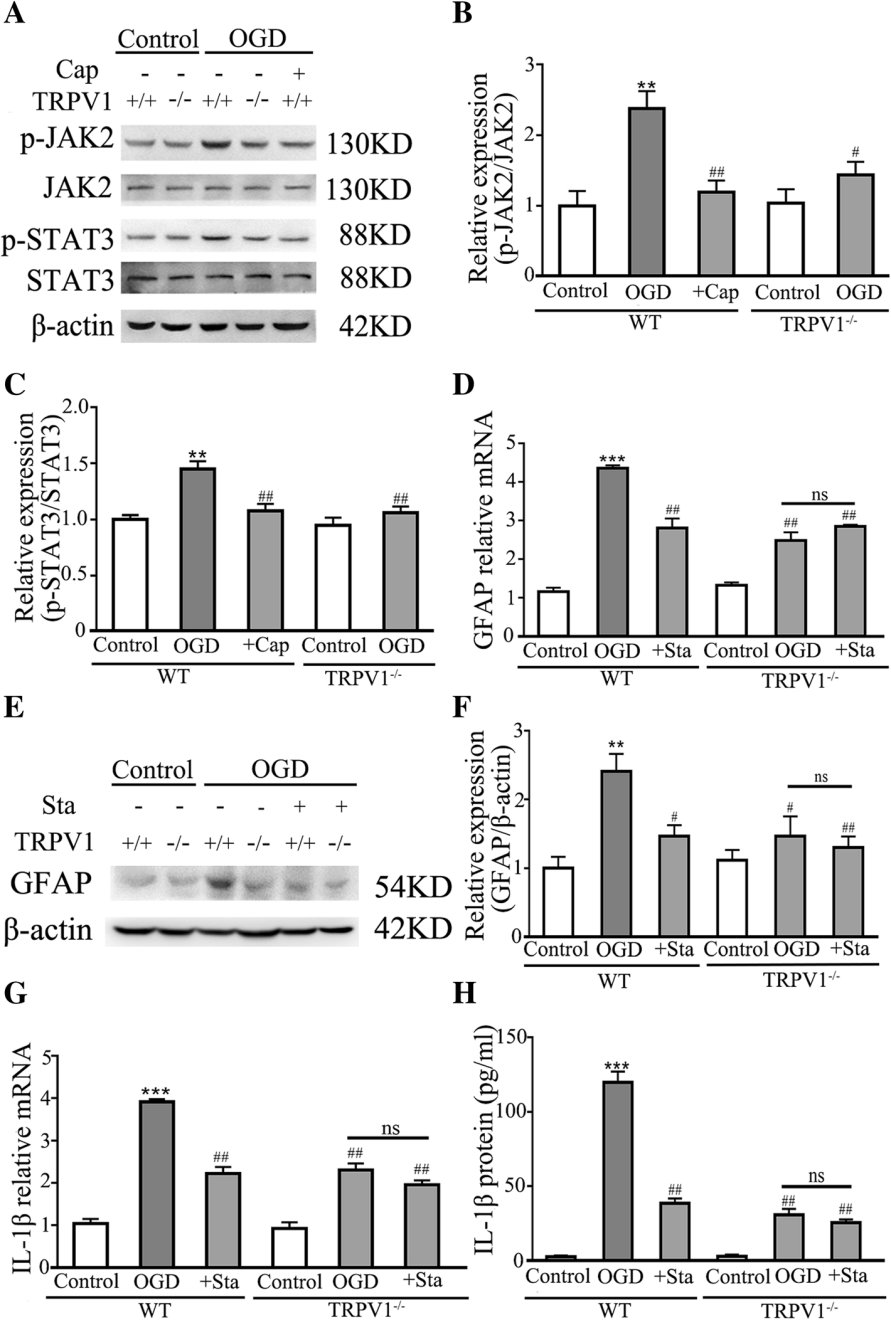
Knocking out TRPV1 inhibited the phosphorylation of JAK2 and STAT3, astrocytes activation, and the expression of IL-1β. **a** Western blot analysis showed the upregulated after OGD, and knocking out TRPV1 or pretreating with capsaicin inhibited the phosphorylation of JAK2 and STAT3 compared to OGD group. **b**, **c** ^#^*P* < 0.05 (Tukey’s test after two-way repeated-measures ANOVA). *n* = 5 for each group. **d** The graphical representation of the fold changes of the expression of GFAP, treating with Stattic (5 μM) inhibited the upregulation of GFAP. *n* = 5 for each group. **e** Western blot revealed treating with Stattic (5 μM) inhibited the upregulation of GFAP. *n* = 5 for each group. **f** As confirmed by quantification of independent blots. ^#^*P* < 0.05 (Tukey’s test after two-way repeated-measures ANOVA). *n* = 5 for each group. **g**, **h** The graphical representation of the fold changes of IL-1β. Treating with Stattic (5 μM) inhibited the upregulation of IL-1β. *n* = 6 for each group. Cap, 10 μM capsaicin. Sta, 5 μM stattic. Average values represent the mean ± SEM. **P < 0.01, ***P < 0.001 versus control (Tukey’s test after one-way ANOVA). ^#^*P* < 0.05, ^##^*P* < 0.01 versus WT-OGD (Tukey’s test after one-way ANOVA). ns *P* > 0.05 (Tukey’s test after one-way ANOVA)

TRPV1 activation has been shown to exacerbate neuro-inflammatory actions by activating pro-inflammatory STAT3 signaling [29]. Thus, Western blot was used to confirm whether TRPV1 activated JAK2-STAT3 signaling at 24 h after OGD. Western blot results showed that phosphorylation of STAT3 and JAK2 were significantly increased after OGD, which was reversed by either genetic ablation or capsaicin-induced desensitization of TRPV1 (Fig. 3a–c), indicating the critical role of TRPV1 on the phosphorylation of JAK2 and STAT3. Next, we used Stattic, a specific inhibitor of STAT3 phosphorylation, to investigate whether pharmacological inhibition of STAT3 phosphorylation attenuates or abolishes TRPV1-induced increase of GFAP expression. Indeed, treating WT astrocytes with Stattic (5 μM) during OGD challenge significantly reduced the expression of GFAP. In marked contrast, Stattic did not inhibit the expression of GFAP in TRPV1 deficiency astrocytes (Fig. 3d–f).

Recent studies have shown that JAK2-STAT3 signaling was vital for the expression of IL-1β in both resident macrophages [51] and CNS microglia [32], and expression of IL-1β has been used to predict the severity of HIE in infants [12]. Therefore, we next verified the role of TRPV1-activated JAK2-STAT3 signaling in OGD-induced IL-1β upregulation in astrocytes. As shown in Fig. 3g, h, mRNA transcripts and protein level of IL-1β were downregulated by inhibiting the phosphorylation of STAT3 in WT astrocytes and Stattic did not inhibit the expression of IL-1β in TRPV1 deficiency astrocytes. These results suggested that TRPV1 promoted the activation of astrocytes and the expression of IL-1β via JAK2-STAT3 signaling after OGD challenge.

### Activation of NLRP3 inflammasome in astrocytes is required for OGD-induced TRPV1-dependent release of IL-1β

**Fig. 4:**
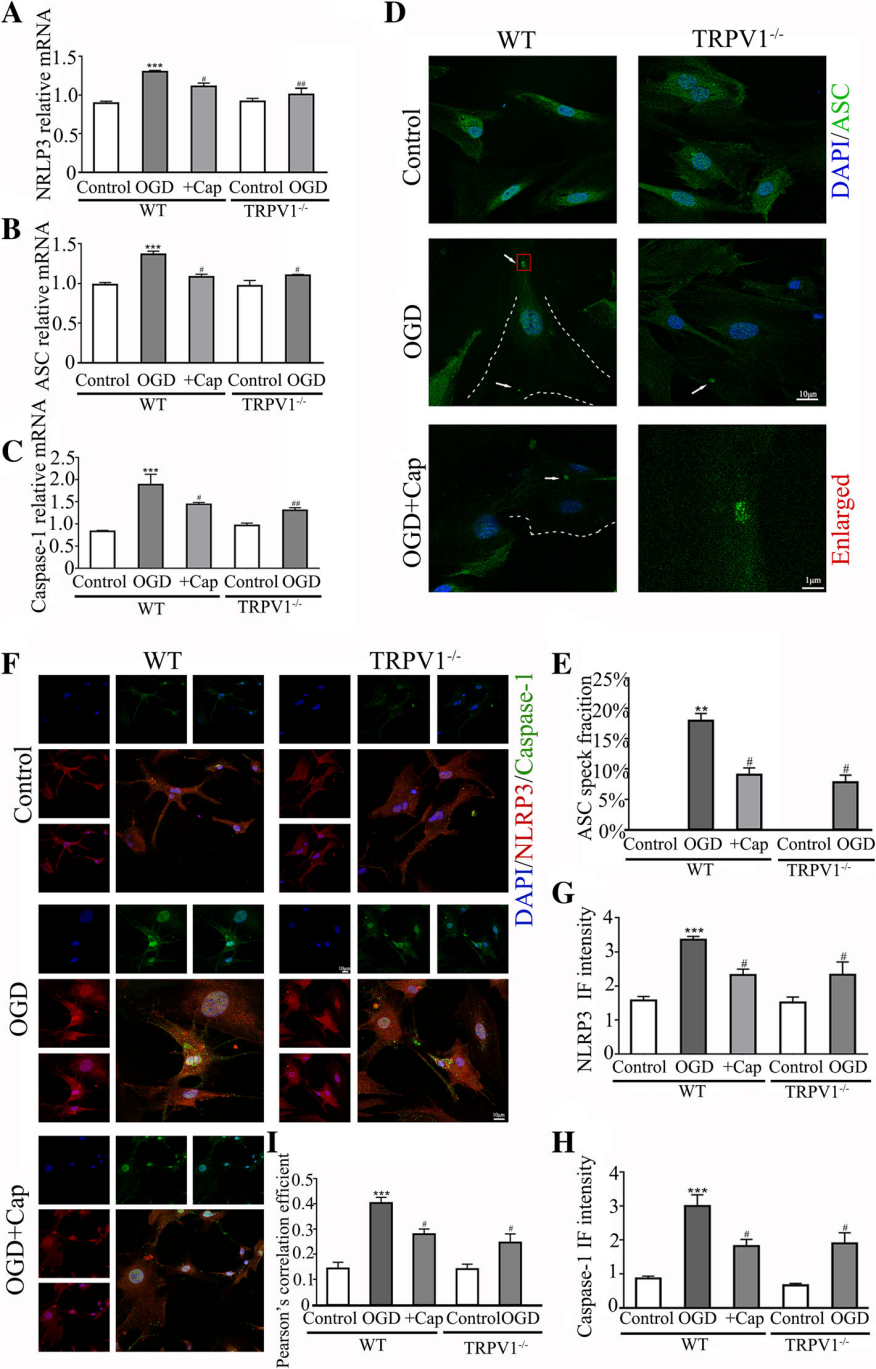
Knocking out TRPV1 attenuates the formation of NLRP3 inflammasome. **a**–**c** The graphical representation of the fold changes of NLRP3, ASC, and caspase-1. Knocking out TRPV1 or pretreating with capsaicin reduced the upregulation of NLRP3, ASC, and caspase-1 compared to OGD group. *n* = 5 for each group. **d** Confocal images showed ASC specks increased after OGD, and knocking out TRPV1 or pretreating with capsaicin inhibited the increase of ASC specks compared to OGD group. White arrows indicate ASC specks and the scale bar = 10 μm, scale bar = 1 μm for the enlarged image. **e** ASC speck positive astrocytes percentage was determined in 10 fields/well and divided by the number of cells counterstained with DAPI. *n* = 4 for each group. **f** Confocal images showed that NLRP3 and caspase-1 were increased after OGD, and knocking out TRPV1 or pretreating with capsaicin inhibited their upregulation compared to OGD group. Scale bar = 10 μm. **g**, **h** The intensity of NLRP3 and caspase-1 fluorescence was determined in 10 fields/well and divided by the number of cells counterstained with DAPI. *n* = 4 for each group. **i** Pearson’s correlation coefficient is shown in the graph from the analysis of independent experiments. *n* = 4 for each group. Cap, 10 μM capsaicin. Average values represent the mean ± SEM. **P < 0.01, ***P < 0.001 versus control (Tukey’s test after one-way ANOVA). ^#^*P* < 0.05, ^##^*P* < 0.01 versus WT-OGD (Tukey’s test after one-way ANOVA)

The activation of NLRP3 inflammasome was critical for IL-1β release in multiple sclerosis (MS), stroke, and Alzheimer’s disease [52]. Following OGD challenge, the levels of mRNA transcripts of NLRP3 inflammasome components (NLRP3, ASC, and caspase-1) increased and the increasing was attenuated by genetic ablation and capsaicin-induced desensitization of TRPV1 (Fig. 4a–c).

ASC is a key adaptor of several inflammasomes including NLRP3 and AIM2, and their activation is reflected by ASC speck assembly or oligomerization [53]. The confocal imaging is used to measure the assembly of ASC speck indicated by the white arrow (Fig. 4d). Upon NLRP3 activation by OGD challenge the dispersed ASC condenses into a large speck in astrocytes, genetic ablation or capsaicin-induced desensitization of TRPV1 reduced ASC oligomerization (Fig. 4d, e). Immunofluorescent staining showed increased intensity (Fig. 4f–h) and co-localization (Fig. 4i) of NRLP3 and caspase-1 after OGD, which were reduced by either genetic ablation of TRPV1 or capsaicin-induced desensitization of TRPV1, suggesting that TRPV1 mediates the release of IL-1β following OGD challenge by activation of NLRP3 inflammasome in astrocytes.

### Brain damage and neurobehavioral disorders induced by neonatal HI are reduced in TRPV1 KO mice

**Fig. 5:**
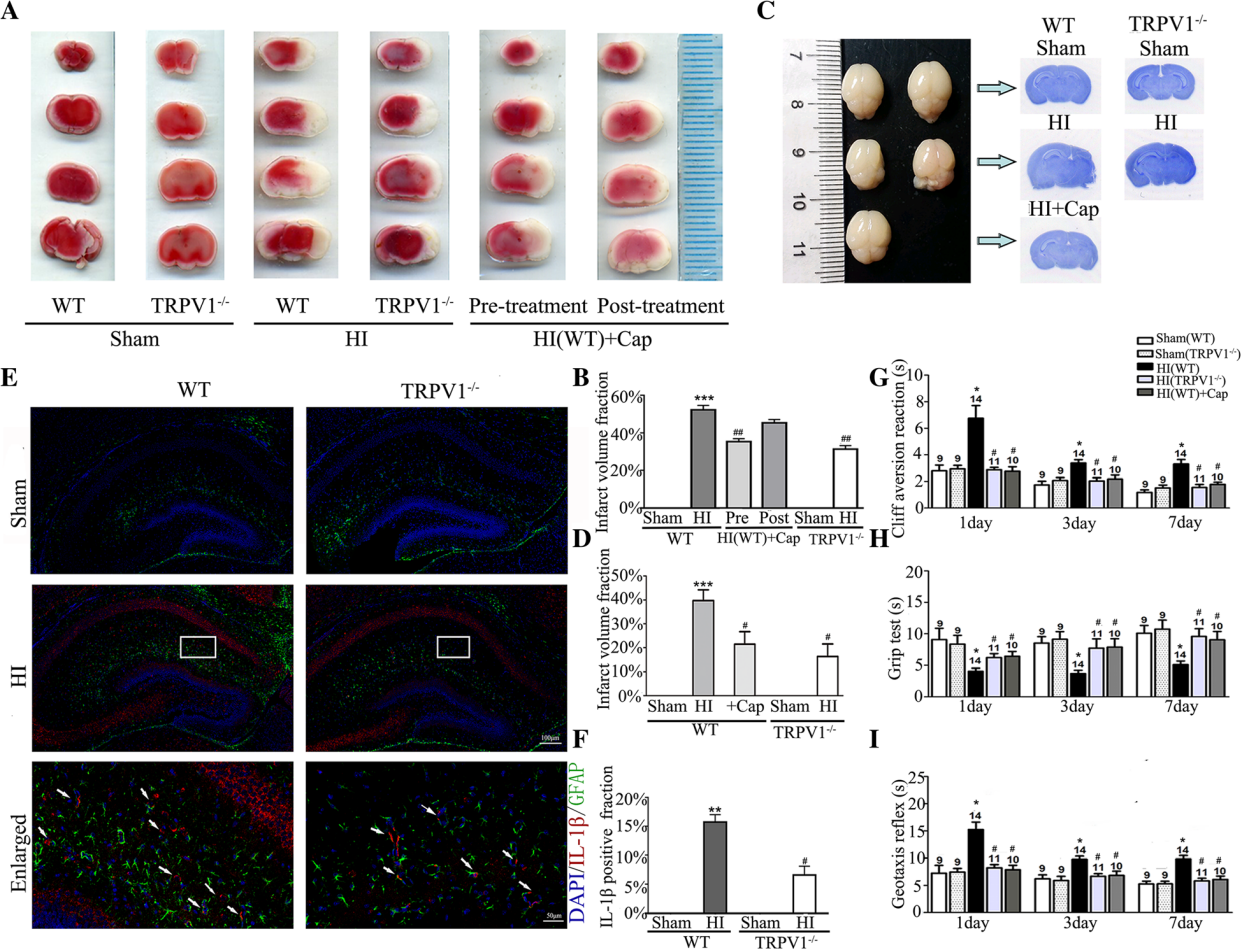
Knocking out TRPV1 reduced infarct volume, brain atrophy, and neurobehavioral loss. **a** Representative TTC stained coronal brain sections from the sham, HI, and capsaicin treatment groups with different injection times as shown, *n* = 7–8 for each group. **b** Quantitative analysis of the infarct volume revealed that knocking out TRPV1 or pretreating with capsaicin reduced the infarct volume. **c** Whole brain Nissl staining from the sham, HI, and capsaicin treatment groups are shown. *n* = 8 for each group. **d** Quantitative analysis of brain atrophy in which knocking out TRPV1 or pretreating with capsaicin produced reduction the infarct volume. *n* = 8 for each group. **e** Confocal images showed knocking out TRPV1 inhibited the increase of IL-1β-positive astrocytes compared to HI group in the ipsilateral hemisphere. White arrows indicate IL-1β-positive astrocytes and the scale bar = 100 μm, scale bar = 50 μm for the enlarged image. **f** IL-1β-positive astrocytes percentage was determined in 10 fields/section and divided by the number of cells counterstained with DAPI. *n* = 4 for each group. **g** The geotaxis reflex results at 1, 3, and 7 days, showing the time that pups took to turn around. *n* = 9–14 for each group. **h** The cliff aversion reaction results at 1, 3, and 7 days, demonstrating the time that pups took to turn their head at the cliff. *n* = 9–14 for each group. **i** The grip test results at 1, 3, and 7 days, showing the time that pups were able to hold onto a horizontal bar. *n* = 9–14 for each group. Cap, 3 mg/kg capsaicin. Average values represent the mean ± SEM. *P < 0.05, **P < 0.01 versus control (Tukey’s test after one-way ANOVA). ^#^*P* < 0.05, ^##^*P* < 0.001 versus WT-HI (Tukey’s test after one-way ANOVA)

IL-1β is the key mediator of neuroinflammation in the CNS [54], and the upper results show that TRPV1 is critical to astrocyte activation and release of astrocyte-derived inflammatory cytokines. To investigate whether the TRPV1-dependent inflammatory response could be translated into brain damage in vivo, we examined brain damage 24 h after neonatal HI in TRPV1 KO mice or WT mice subjected to capsaicin pretreatment. As shown in Fig. 5a, b, the TTC-negative area in the ipsilateral hemisphere was significantly decreased in TRPV1 KO mice and WT mice pretreated with capsaicin (intraperitoneal injection 3 mg/kg 0.5 h before neonatal HI). In marked contrast, post-treating with capsaicin (3 mg/kg, administered 5 min after neonatal HI) did not effectively reduce the TTC-negative area in WT mice (Fig. 5a, b). Next, we used whole-brain Nissl staining to determine the role of TRPV1 in brain atrophy 24 h post neonatal HI (Fig. 5c, d). Neonatal HI produced marked brain atrophy compared with the ipsilateral side of the sham group. Brain atrophy in either TRPV1 KO mice or WT capsaicin pre-treatment mice is significantly reduced. Immunofluorescence showed that the number of IL-1β-positive astrocytes in the ipsilateral hemisphere hippocampus (indicated by the white arrow) decreased in TRPV1 KO mice compared with WT mice after neonatal HI (Fig. 5e, f).

To investigate whether TRPV1 is involved in neonatal HI-induced neurobehavioral disorders, three different neurobehavioral tests including cliff aversion reaction (Fig. 5g), grip test (Fig. 5h) and geotaxis reflex (Fig. 5i) were used. Neonatal HI significantly prolonged the time for WT pups to turn around in the cliff aversion reaction, reduced the holding time in the grip test, and increased the head-turning time in the geotaxis reflex at days 1, 3, and 7 compared with the sham group, which was recovered in TRPV1 KO and WT capsaicin pre-treatment mice. These results suggest that TRPV1 contributes to brain atrophy, infarct size, and loss of reflexes and motor ability caused by neonatal HI.

## Discussion

Both astrocytes and microglia were activated after injury by pro-inflammatory mediators, cytokines, and ROS that are secreted by injured neurons and glial cells [55]. The role of microglia in HIE was well established and microglial activation and aggregation had become one of the hallmarks for HIE in human infants [3, 56–58]. Notably, astrocytes are also involved in the pathogenesis of neurological diseases and sterile inflammatory responses [4, 9, 59] and the role of astrocytes in CNS inflammatory was controversial (both detrimental and beneficial) in brain ischemia [3]. Early studies have extensively focused on the protective effects of astrocytes, such as the isolation of the damaged area from the rest of the CNS tissue and reconstruction of the compromised blood-brain barrier [60, 61]. Recent studies, however, demonstrated that astrocytes have a detrimental role in response to ischemic stress [4, 10, 62–64].

Ion channels (kv4.1, kv1.3, TRPV1, TRPM7, NKCC1…) and intracellular ion homeostasis (K^+^, Na^+^, Ca^2+^, and Cl^−^) are critical for glia proper functions in CNS [65–67]. TRPV1 that is mainly permeable to Ca^2+^ naturally expresses in the endomembrane system of microglia and astrocytes which plays important roles in astrocyte and microglial activation [68–70]. It has been proved that overactivated TRPV1 increase NADPH oxidase-mediated reactive oxygen species (ROS) generation [71, 72], mitochondrial disruption [73] in microglia, and microglia-induced inflammation [74]. TRPV1 also accelerate astrocyte and microglial migration and chemotaxis during stress by directly interacting with cytoskeletal elements [75, 76]. The role of TRPV1 in microglia is well explored [26], while its distinctive function in astrocyte is not fully understood in a variety of neuroimmune diseases.

**Fig. 6:**
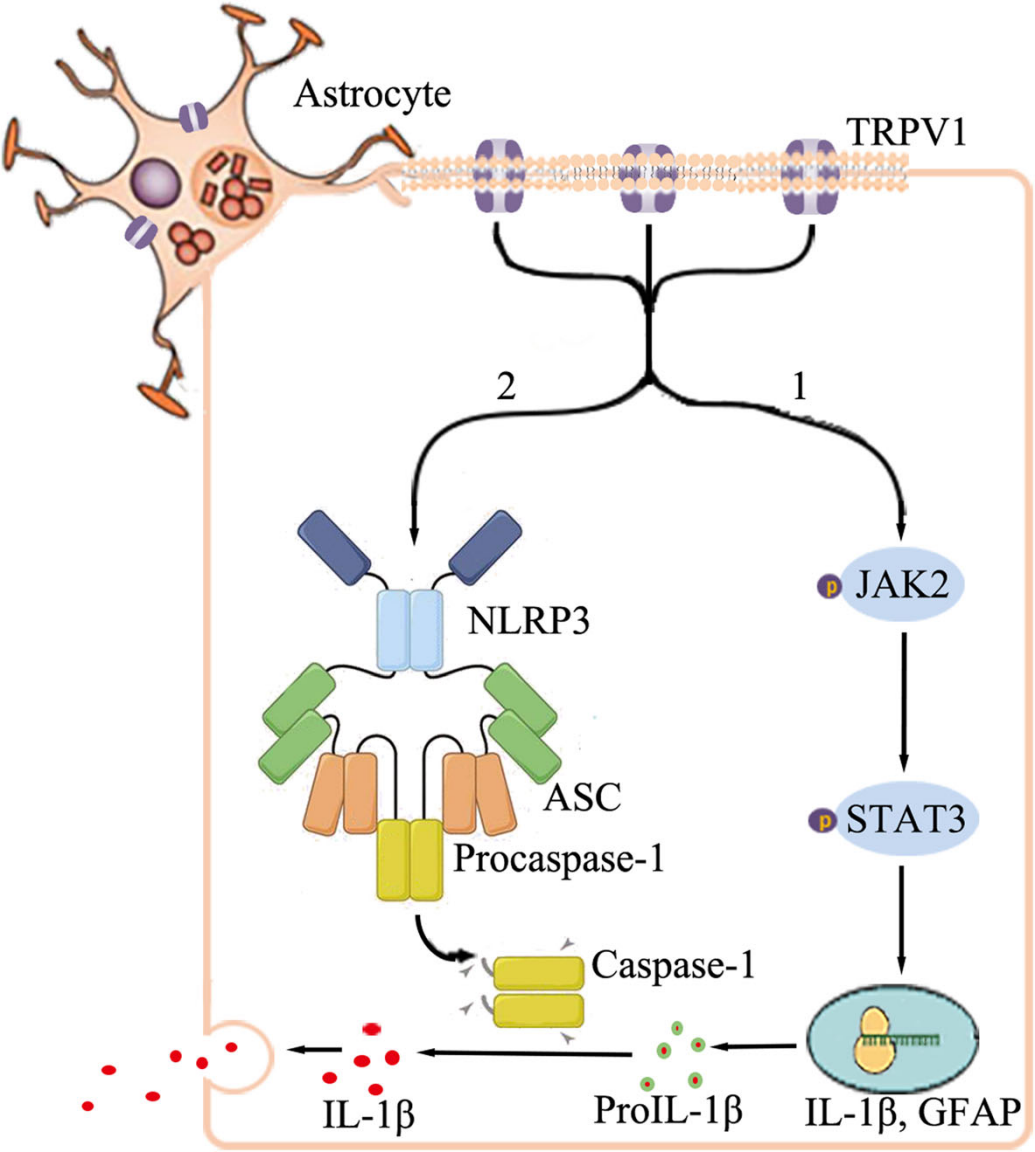
Schematic illustration of the proposed mechanism. TRPV1 plays an important role in neonatal HI-induced astrocytes activation and the transcription and post-translational protein modification of IL-1β. (1) TRPV1 stimulated the phosphorylation of JAK2, and phosphorylated JAK2 promoted the phosphorylation of STAT3. And activated STAT3 bound to its target genes (GFAP and IL-1β) and upregulated their expression. (2) TRPV1 promoted the formation of NLRP3 inflammasome (NLRP3, ASC, and caspase-1), which mediated the activation of caspase-1 and then stimulated the cleavage of pro-IL-1β

In the present study, we showed that during the subacute phase of HI (24 h), astrocyte and microglia were overactivated and microglia activation is about 3 h earlier than astrocyte. This may because microglia acting as the constant sensors of changes in the CNS microenvironment is more sensitive to insults [77]. TRPV1, which is activated after ischemia [78] intensely mediate astrocyte activation compared to microglia, increases IL-1β release and exacerbates ischemia-induced brain damage (brain atrophy, infarct size, and neurobehavioral loss). TRPV1 is required for the phosphorylation of JAK2 and STAT3 which promotes the transcription of GFAP and IL-1β. TRPV1 function is critical for the activation of NLRP3 inflammasome which promotes the cleavage of pro-IL-1β (Fig. 6). Our studies reveal a previously unknown function of astrocytic TRPV1 and potentially identify a critical target for the treatment of CNS inflammation.

Injured cortex becomes hypertrophic in many types of CNS disorders such as stroke and neurotrauma, which is often accompanied with the upregulation of GFAP in astrocytes [9]. As expected, we found that GFAP expression increased in a time-dependent manner after neonatal HI, which is mediated by TRPV1 as either genetic ablation or capsaicin-induced systemic desensitization of TRPV1 inhibited the upregulation of GFAP. In vitro studies further confirmed that TRPV1 directly regulates the function of astrocytes in a cell autonomous manner without the requirement of neurons or microglia, since inhibition of TRPV1 function prevented astrocytes from cellular edema, reduction of branch processes, cell death, and excessive activation caused by OGD challenge.

The role of activated astrocytes has been reported in the sterile inflammatory response and the release of several cytokines, such as IL-1β, IL-6, and TNF [79] in middle cerebral artery occlusion (MCAO). Our previous work demonstrated that TRPV1 activation promotes the expression of TNF, IL-1β, IL-6, and high mobility group box 1 (HMGB1) in repetitive febrile seizure (FS) mice [36]. Consistent with these observations, we found that TRPV1 is required for OGD-induced upregulation of IL-1β, IL-6, and TNF in astrocytes. Interestingly, IL-10 mRNA transcript was slightly increased in a TRPV1-dependent manner after neonatal HI, which might be caused by a negative feedback from the astrocytes induced by severe inflammatory responses [80, 81].

How does TRPV1 mediate astrocyte activation and upregulation of pro-inflammatory cytokines? Previous researches demonstrated that the JAK2-STAT3 pathway is involved in the activation of astrocytes and the expression of cytokines both in vivo and in vitro [30–32]. Moreover, in inflammatory signaling cascades in the brain, TRPV1 has been shown to exacerbate neuro-inflammatory actions by activating pro-inflammatory STAT3 signaling [29]. Inhibition STAT3 phosphorylation via JAK2-blockade reduces neonatal HI-induced neuroinflammation and tissue loss [82]. Consistent with these studies, we found that TRPV1 promoted the phosphorylation of JAK2 and STAT3 induced by OGD challenge. Moreover, a specific inhibitor of STAT3 (Stattic) significantly reduced the expression of GFAP and IL-1β following OGD challenge in a TRPV1-dependent manner. These results suggest that TRPV1 mediates the activation of astrocytes and the expression of IL-1β via the JAK2/STAT3 pathway.

IL-1β in umbilical and peripheral blood is noteworthy upregulated in children suffered from HIE, and its expression level corresponds to the grades and adverse outcomes of HIE [83]. The release of IL-1β depends on the activation of NLRP3 inflammasome which cleaves the pro-inflammatory cytokines IL-1β to its active form by mediating the activation of caspase-1 [15, 84, 85]. It has been proved that, in other disease models such as intracerebral hemorrhage model, ischemia/reperfusion injury model, and hemorrhage transgenic amyotrophic lateral sclerosis model, NLRP3 inflammasome is activated in astrocytes [16–19]. In the present study, the activation of NLRP3 inflammasome followed by IL-1β releasing increased after OGD challenge. And inhibition of TRPV1 blocked this modification, suggesting that TRPV1 drives the release of IL-1β by stimulating the activation of NLRP3 inflammasome after OGD in astrocytes.

In vivo, hampered TRPV1 function significantly reduced brain atrophy, infarct size, and the number of IL-1β-positive astrocytes in the hippocampus. Additionally, the recovery of neurobehavioral disorders indicated by three independent neurobehavioral developmental tests (the geotaxis reflex, cliff aversion reaction, and grip test) after neonatal HI was potentiated in TRPV1 KO mice. Our results suggest that astrocytic TRPV1 is required for the exacerbation of the brain damage and neurobehavioral disorders after neonatal HI. Of note, since TRPV1 do express by microglia/macrophages, neuron [78], and neutrophils [86], these types of cells might contribute to the detrimental effects mediated by TRPV1 independently or cooperatively in HIE. Future studies using cell-specific TRPV1 knockdown/knockout mice needed to confirm this possibility.

## Conclusion

TRPV1 promotes activation of astrocytes and release of astrocyte-derived IL-1β mainly via JAK2-STAT3 signaling and activation of the NLRP3 inflammasome. Our findings provide mechanistic insights into TRPV1-mediated brain damage and neurobehavioral disorders caused by neonatal HI and potentially identify astrocytic TRPV1 as a novel therapeutic target for treating HIE in the subacute stages (24 h).

## Additional file

Additional file 1: Knocking out TRPV1-reduced GFAP and Iba-1-positive cell in hypoxia-ischemia brain tissue. (A) GFAP and Iba-1-positive cell were examined in the ipsilateral hemisphere sections from Sham and HI groups. Scale bar = 100 μm. (B, C) The number of GFAP and Iba-1-positive cell in the ipsilateral hemisphere hippocampus. *n* = 6 for each group. Average values represent the mean ± SEM. *P < 0.05, **P < 0.01, ***P < 0.001 (Tukey’s test after one-way ANOVA and two-way ANOVA). (TIF 8629 kb)

## Abbreviations

HI: Hypoxia-ischemia
TRPV1: Transient receptor potential vanilloid 1
GFAP: Glial fibrillary acidic protein
Iba1: Ionized calcium-binding adapter molecule 1
JAK2: Janus kinase 2
STAT3: Signal transducer and activator of transcription 3
NLRP3: Nucleotide-binding, oligomerization domain (NOD)-like receptor pyrin domain-containing protein 3
ASC: Apoptosis-associated speck-like protein containing carboxyl-terminal CARD
HIE: Hypoxic-ischemic encephalopathy
FS: Febrile seizure
DAPI: 4:6-Diamidino-2-phenylindole
IL-1β: Interleukin 1β
IL-6: Interleukin 6
OGD: Oxygen-glucose deprivation
PBS: Phosphate-buffered saline
TNF: Tumor necrosis factor
IL-10: Interleukin 10
TTC: 2, 3, 5-Triphenyltetrazolium chloride monohydrate
PMSF: Phenylmethanesulfonyl fluoride
PVDF: Polyvinylidene fluoride
BSA: Bovine serum albumin
ROS: Reactive oxygen species
