# Supplementary figures and images for: TRPV1 mediates astrocyte activation and interleukin-1β release induced by hypoxic ischemia (HI)
Source: J Neuroinflammation. 2019 May 29;16:114. doi: 10.1186/s12974-019-1487-3 (PMC6540554; doi:10.1186/s12974-019-1487-3)

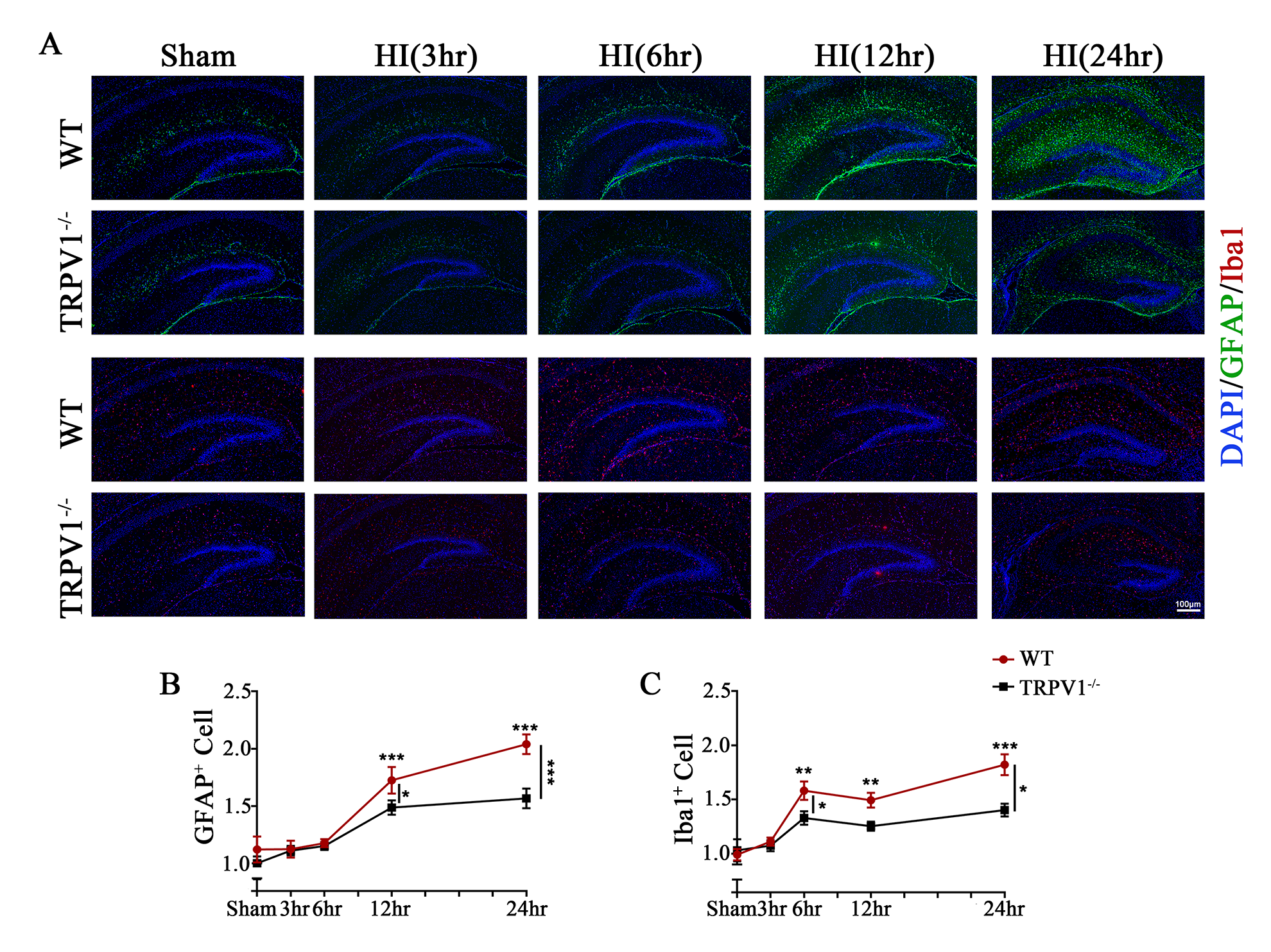

Supplement: Supplementary file 1 — Knocking out TRPV1-reduced GFAP and Iba-1-positive cell in hypoxia-ischemia brain tissue. (A) GFAP and Iba-1-positive cell were examined in the ipsilateral hemisphere sections from Sham and HI groups. Scale bar = 100 μm. (B, C) The number of GFAP and Iba-1-positive cell in the ipsilateral hemisphere hippocampus. n = 6 for each group. Average values represent the mean ± SEM. *P < 0.05, **P < 0.01, ***P < 0.001 (Tukey’s test after one-way ANOVA and two-way ANOVA). (TIF 8629 kb) [file 12974_2019_1487_MOESM1_ESM.tif]
